# A Challenging Case of an Ectopic Cushing Syndrome

**DOI:** 10.1155/2014/413136

**Published:** 2014-11-09

**Authors:** Joana Menezes Nunes, Elika Pinho, Isabel Camões, João Maciel, Pedro Cabral Bastos, Conceição Souto de Moura, Paulo Bettencourt

**Affiliations:** ^1^Endocrinology, Diabetes and Metabolism Department, Centro Hospitalar São João, Rua Alberto Serpa No. 150/152, 4100-010 Porto, Portugal; ^2^Faculty of Medicine, Porto University, Alameda Professor Hernâni Monteiro, 4200-319 Porto, Portugal; ^3^Internal Medicine Department, Centro Hospitalar São João, Alameda Professor Hernâni Monteiro, 4200-319 Porto, Portugal; ^4^Cardiothoracic Surgery Department, Centro Hospitalar São João, Alameda Professor Hernâni Monteiro, 4200-319 Porto, Portugal; ^5^Pathology Department, Centro Hospitalar S. João, Alameda Professor Hernâni Monteiro, 4200-319 Porto, Portugal

## Abstract

Bronchopulmonary carcinoids are rare pulmonary neoplasms although they account for most cases of ectopic ACTH syndromes. When feasible, the mainstay treatment is surgical resection of the tumor. We report the case of a 52-year-old woman with signs and symptoms suggestive of hypercortisolism for 12 months, admitted to our department because of community acquired pneumonia. Blood hormone analysis showed increased levels of ACTH and urinary free cortisol and nonsuppressibility to high- and low-dose dexamethasone tests. Pituitary MRI showed no lesion and no central-to-peripheral ACTH gradient was present in bilateral inferior petrosal sinus sampling. CRH stimulation test suggested an ectopic ACTH source. Thoracic CT scan revealed a nodular region measuring 12 mm located in the inferior lingular lobule of the left superior lung with negative uptake by ^18^-FDG-PET scan and negative SRS. The patient was successfully treated with an atypical lung resection and histology revealed an atypical bronchial carcinoid tumor with positive ACTH immunoreactivity. This was an interesting case because the patient was admitted due to pneumonia that may have been associated with her untreated and chronic hypercortisolism and a challenging case of ectopic ACTH syndrome due to conflicting results on the diagnostic exams.

## 1. Introduction

Cushing syndrome (CS) refers to a diverse symptom complex resulting from excess steroid hormone production by adrenal gland (endogenous) or from sustained administration of glucocorticoids (exogenous) [1, 2]. The endogenous CS comprises three distinct pathogenic disorders: pituitary, adrenal, and ectopic [1]. Ectopic Cushing syndrome (ECS) results from autonomous ACTH production from extrapituitary malignancies with elevated plasma levels of ACTH accounting for 15% of cases of CS [2].

Although numerous malignancies have been associated with this disorder, bronchogenic carcinoma accounts for most cases [1–5]. Bronchial carcinoids are neuroendocrine tumors arising from neuroendocrine Kulchitsky cells, located in the bronchial epithelium. Typical carcinoid is a slow growing neoplasm (<2 mitoses/10 HPF, lacking necrosis) but there are some poor (small cell lung) and undifferentiated neuroendocrine carcinomas. Atypical carcinoid (2–10 mitoses/10 HPF, with necrosis) has an intermediate behavior [6]. All of them share certain morphologic and biochemical characteristics such as the capacity to synthesize neuropeptides, being identified by immunohistochemical markers such as synaptophysin, neuron-specific enolase (NSE), and chromogranins [7, 8].

The common clinical features of classical CS include central obesity, hypertension, glucose intolerance/diabetes mellitus, plethoric facies, purple striae, hirsutism, oligomenorrhea/amenorrhea, muscle weakness, bruising, and osteoporosis. Less common features are mental changes, hyperpigmentation, acne, and hypokalemic alkalosis and more subtle presentations may be encountered in early stages or with mild hypercortisolemia such as moderate weight gain, diabetes, metabolic syndrome, or osteoporosis [1–3, 9].

Hypercortisolism due to ectopic ACTH syndrome is usually severe and of rapid onset [1]. Chronic and severe excess of cortisol levels is associated with life-threatening infections [10] and most patients with bronchial carcinoids present with cough, hemoptysis, or recurrent chest infections [11]. It is to understand that the presence of both entities severely compromises clinical outcome.

Since the treatment of choice for ectopic ACTH syndrome (EAS) is complete resection of the tumor, the correct localization and confirmation of the ectopic ACTH source play the key role. It has been reported that up to 50% of ECS are undetectable by CT and MRI [12]. However, it is often difficult to localize an occult and indolent tumor by conventional imaging tests, especially bronchial carcinoid. Approximately 80% and 60% of typical and atypical bronchial carcinoids, respectively, express somatostatin receptors and recent data suggest that SRS is one of the exams used for tumor localization [13–15]. ^18^-FDG-PET scan offers higher special resolution than SRS, improving sensitivity for detection of small lesions, but its result is dependent on the tumor metabolism [16–18].

When diagnostic exams are inconclusive in locating the tumor, symptoms and signs are commonly present for several months and are associated with worse outcome [2].

We describe a challenging case of EAS with discordant results on the endocrine tests and imagiology.

## 2. Case Report

A 52-year-old woman was admitted to our department because of cough and dyspnea with type 1 respiratory insufficiency. Etiological investigation revealed a community acquired pneumonia and the patient was successfully treated with 1 g of ceftriaxone for 10 days.

The medical history was remarkable for hypertension diagnosed at the age of 40 years treated with low salt ingestion and no regular medication until one year before. Since then, she complained of progressive asthenia and edema (especially of the face, neck, and supraclavicular region), centripetal obesity, abdominal striae, hair loss, amenorrhea, weight gain (25 Kg), and depressive symptoms. She denied any history of taking exogenous steroids or herbal medicine. Familial clinical history was irrelevant.

**Figure 1:**
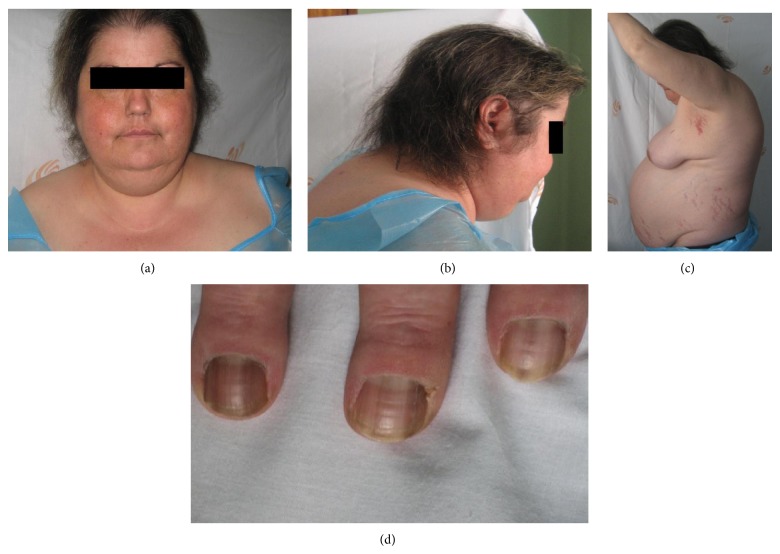
Patient phenotype.

On physical exam, the patient presented Cushingoid features (moon facies, multiple telangiectasias on the face and thorax, skin atrophy, buffalo cervix, centripetal obesity and extended striae on the abdomen and arms, distal muscular atrophy, and peripheral edema) (Figure 1). Blood pressure was high (158/90 mmHg), cardiac and pulmonary auscultations were normal, and there were no abdominal palpable masses. Liver was palpable 3 cm below right thoracic cage.

During hospital stay, routine laboratory results showed hypercholesterolemia (total cholesterol 2.70 g/L, HDL cholesterol 0.82 g/L, LDL cholesterol 1.71 g/L, and triglycerides 1.52 g/L), elevated fasting plasma glucose 1.32 g/L with HbA1c 5.8% (hemoglobin = 13.4 g/dL), and hypokalemia (K+ = 3.2 mEq/L) with no other electrolyte disturbances and no renal/liver dysfunction.

As clinical suspicion of endogenous hypercortisolism was high, screening for CS was decided. Hormonal tests revealed markedly increased 24 h urinary free cortisol excretion on two consecutive days, elevated morning plasma ACTH and cortisol levels, and lack of ACTH/cortisol circadian rhythm (Table 1).

Low-dose and high-dose dexamethasone suppression tests were performed with no suppression of serum cortisol or urinary free cortisol (Table 2). Urinary 5-hydroxyindoleacetic acid (5-HIAA) was normal.

To identify a potential pituitary lesion, a pituitary MRI was performed but there was no identifiable tumor and the bilateral inferior petrosal sinus sampling (BIPSS) showed no central-to-peripheral ACTH gradient (Table 3).

At this point, performing a peripheral corticotropin-releasing hormone (CRH) stimulation test was decided to differentiate Cushing disease (CD) from ectopic ACTH production, although it offers 86% sensitivity and 90% specificity for pituitary CS [19]. CRH stimulation test suggested an ectopic ACTH source, since ACTH and cortisol responses were flat after CRH administration (Table 4).

Plain chest X-ray showed no other abnormalities besides the pneumonia ones. Bronchoscopy and cytological examination of bronchoalveolar lavage were negative for malignancy.

**Figure 2:**
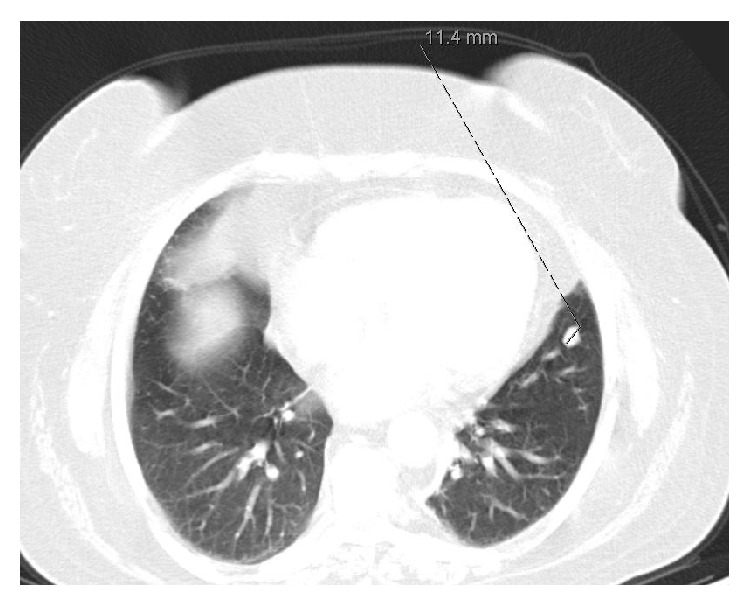
Chest CT scan.

Chest CT scan revealed one nodular defined lesion in the inferior lingular lobule of the left superior lung, measuring 12 mm (Figure 2). Abdominal CT scan showed normal adrenal glands (no signs of hyperplasia, enlargement, or nodules) and no other suspicious lesions.

**Figure 3:**
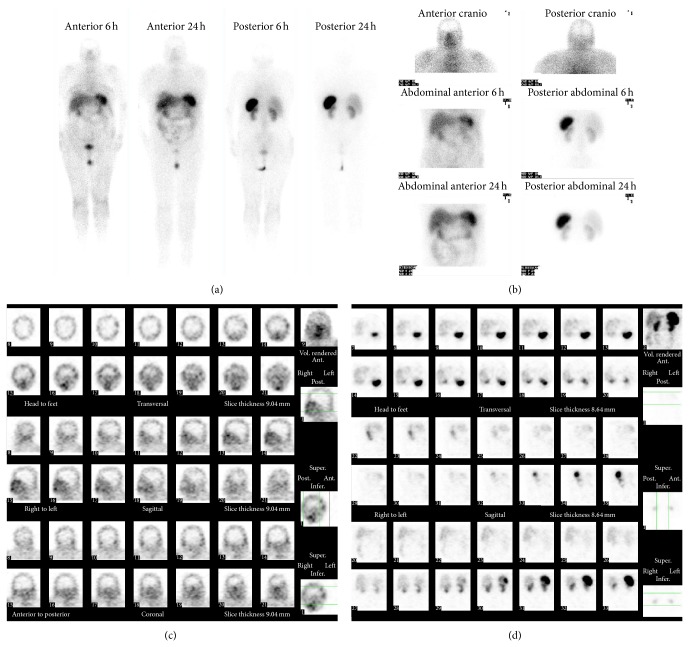
Somatostatin receptor scintigraphy (^111^-indium-pentetreotide).

To confirm that the pulmonary lesion was the ectopic ACTH source, performing a SRS (^111^-indium-pentetreotide) was decided but no lesion with somatostatin receptors was identified (Figure 3).

At this time, the patient was successfully treated with intravenous antibiotics for pneumonia and so was discharged with losartan 100 mg id plus amlodipine 10 mg id to control blood pressure, oral potassium tablets to correct hypokalemia, and ketoconazole 1200 mg id to control hypercortisolism and was referred to consultation to localize the ectopic ACTH source.

Three months after hospital discharge, chest CT scan confirmed the presence of a stable pulmonary nodule. Two thoracic biopsies were performed but there was insufficient material for diagnosis.

The digestive endoscopic study was normal.

^18^-FDG-PET CT scan showed no uptake at all, namely, in the suspicious pulmonary lesion. Bone scan was normal.

These discordant results from the endocrine and imaging tests suggested that the pulmonary lesion could be the etiology of the ACTH syndrome.

**Figure 4:**
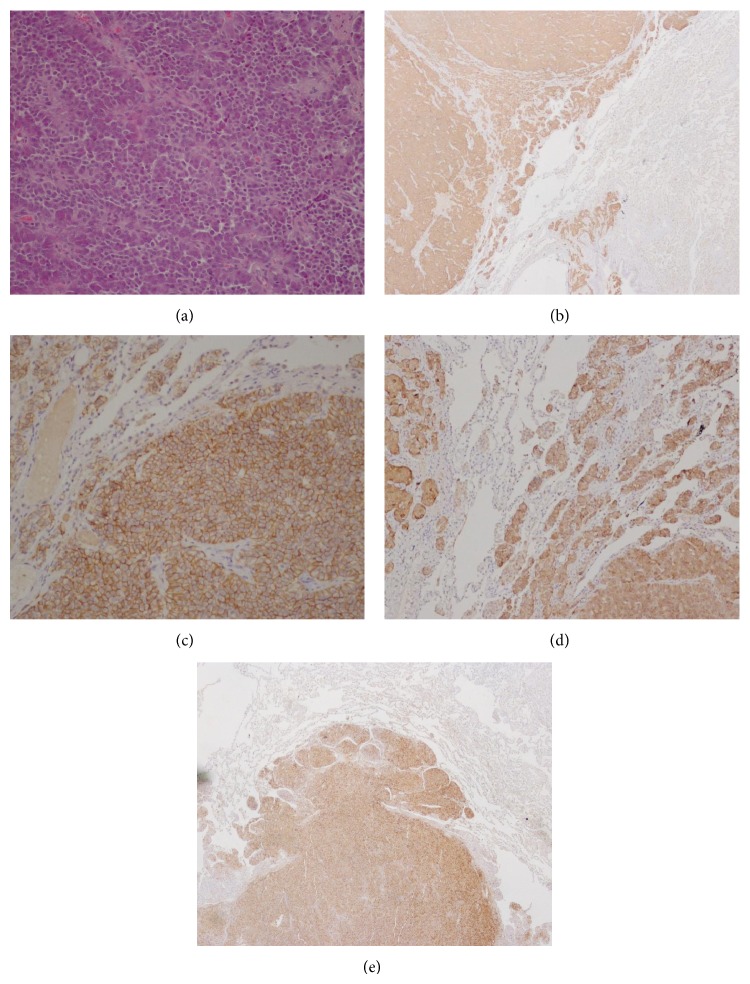
Histology. (a) Hematoxylin-eosin staining, ×10: organoid and trabecular pattern. (b) Positive immunoreactivity to chromogranin. (c) Positive immunoreactivity to synaptophysin. (d) Positive immunoreactivity to NCAM. (e) Positive immunoreactivity to ACTH.

After obtaining informed consent from the patient, she underwent thoracic surgery with atypical pulmonary resection. Histology of the resected lung tumor revealed a bronchial atypical carcinoid tumor with 4 mitoses/10 HPF (Figure 4) and positive immunostainings for ACTH and other neuroendocrine tumor markers (chromogranin A, synaptophysin) without somatostatin receptors.

Postoperative course was uneventful. After surgical removal, plasma ACTH and cortisol levels became normalized and the patient experienced marked alleviation of her symptoms. Actually, five years after surgery, she is free from tumor recurrence and hypercortisolism.

## 3. Discussion

ECS frequently presents a major diagnostic challenge [5–7, 11, 16] and is associated with diagnostic pitfalls despite several and extensive diagnostic exams [8].

The ectopic source of ACTH is located in the lungs in over 45% of tumors and most of the cases (>25%) are bronchial carcinoid tumors [4, 20].

Usually, patients present hypertension, asthenia, and hypokalemia at diagnosis [5] and commonly report a long duration of CS symptoms before surgical resection, most likely reflecting the rarity of this condition and the paucity of pulmonary symptoms [2], delaying attempt at diagnosis. In our case, the patient presented CS for 12 months but did not seek medical advice.

Diagnosing EAS is difficult [21, 22] as none of the dynamic endocrine tests achieves 100% accuracy [5]. According to Ilias et al. [23], 21% and 26% of patients with EAS have false-positive responses to dexamethasone and/or CRH stimulation test and in their experience BIPSS is the best test for diagnosis. Recent data also suggest BIPSS to be the most accurate and reliable method to differentiate CD from EAS with a sensitivity of 88–100% and a specificity of 90–100% [5, 24, 25].

In our case, dynamic biochemical tests were discordant since (a) the suppression of serum cortisol in high-dose dexamethasone test was >50% from baseline suggesting CD, (b) the increase in serum cortisol and plasma ACTH after CRH stimulation was less than 20% and 35%, respectively, and (c) there was no pituitary lesion and no central-to-peripheral gradient on BIPSS, both suggesting ECS.

The rationale for using high-dose dexamethasone suppression test to differentiate CD from ECS is based on the principle that pituitary tumors are only partially autonomous maintaining feedback mechanism at higher set point than normal while ectopic ACTH tumors are usually autonomous and therefore cannot be suppressed [19]. However, some ectopic tumors may be suppressible by high doses (especially bronchial carcinoids that may present an anomalous behavior [6, 9, 11]) and pituitary macroadenomas may be not [19]. Given our results, we decided to search for an ectopic ACTH production.

Imaging studies are the cornerstone of EAS management, because surgical removal of the tumor is the only potential curative treatment [5, 8, 20, 21]. It has been described that, in 30% to 50% of the patients with ACTH dependent CS, the ACTH source is not identified by conventional imaging modalities such as CT, PET, MRI, endoscopic ultrasound, and SRS [25]. Other series also report that, despite extensive evaluation, EAS identification is not achieved between 12% and 19% [23].

However, once suspected, CT remains the preferred modality in localizing pulmonary carcinoids [26] and in our case it did identify a pulmonary lesion. To confirm that this lesion was the ectopic ACTH source and as SRS-octreoscan has been suggested as a diagnostic tool and second-line therapy in some cases [27], performing a SRS was decided but we found no suspicious lesion. Typical carcinoid tumors usually have high concentration of somatostatin receptors among which octreotide binds with higher affinity to SSTR-2 and SSTR-5 [25]. Usually the lesion size detection limit for an octreoscan is 5 mm [8]. According to Zemskova et al. [24] octreotide scan presents a sensitivity of 57% and a positive predictive value of 79%. The expression pattern of SSTR subtype in bronchial carcinoid tumors has not yet been extensively investigated. In the present case, the lack of somatostatin receptors made tumor identification difficult and in this particular case treatment with somatostatin antagonists would be ineffective.

^18^-FDG-PET is a second-line diagnostic procedure when other imaging modalities fail to identify the cause of EAS [28]. It has a sensitivity of 64% and a positive predictive value of 53% [24, 25]. ^18^-FDG-PET is known to identify tumors with high proliferative activities [25] and so it is reasonable to assume that our patient's bronchial carcinoid seemed metabolically not active.

The optimal management of ectopic ACTH secretion secondary to an underlying malignancy is surgical resection of the tumor. However, until surgical procedure, medical therapy for CS is often necessary and in our case the patient was discharged on ketoconazole therapy for steroidogenesis inhibition [29, 30]. Mitotane, ketoconazole, aminoglutethimide, and metyrapone are drugs used to reduce cortisol [29, 30]. Etomidate is an intravenous short-acting anesthetic drug that can immediately decrease adrenal steroid production and so can be used for acute control of severe hypercortisolism [30]. Data suggest that its use is safe and effective in patients unable to take oral medications [31].

In our patient, given the scenario of EAS and chronic life-threatening hypercortisolism for more than one year with its associated morbidity and as there was no other evident source for the ACTH production, a multidisciplinary team, together with the patient, decided on surgical removal of the suspected pulmonary lesion. Performing a Ga-68 DOTATOC-PET scan could have been decided since recent reports [32] and studies [15] suggest this imaging tool to be a promising new modality for EAS localization.

Actually, five years after surgery, the patient has no clinical or biochemical evidence of recurrence.

In summary, EAS diagnosis is quite challenging, especially when endocrine tests and imaging modalities are conflicting and inconclusive. We presented an interesting case of EAS with conflicting results on dynamic biochemical tests and inconclusive imaging exams. The only identifiable source of ectopic ACTH production was a pulmonary lesion measuring 12 mm identified only on CT scan with no uptake by ^18^-FDG-PET and negative SRS. Besides underscoring how difficult it is to identify and localize the ectopic ACTH source, this case also points out that chronic hypercortisolism may be present for some time before diagnosis and is responsible for increased morbidity and mortality.

## Figures and Tables

**Table 1:** Hormonal tests.

Hormonal tests	Day 1		Day 2		Reference values
	8 a.m.	16 p.m.	8 a.m.	16 p.m.	
Cortisol	41.6	43.9	47.7	46.8	6.2–19.4 ug/dL
ACTH	209.0	212.0	140	55.6	<46 pg/mL
24 h-urinary free cortisol	1706.0		1522.5		36–137 ug/day
Midnight salivary cortisol	—		9.3		<2.0 ng/mL

**Table 2:** Low- and high-dose dexamethasone suppression tests.

	Low-dose dexamethasone test			High-dose dexamethasone test		End
	Baseline	Day 1	Day 2	Day 3	Day 4	Day 5
Cortisol 8 a.m.	35.4	47.7	28.0	24.6	10.4	6.0
Cortisol 16 p.m.	35.4	34.1	25.3	14.5	7.8	—
ACTH 8 a.m.	86.6	238.0	136.0	144.0	102.7	76.5
ACTH 16 p.m.	182.0	163.0	55.3	86.9	64.7	—
24 h urinary free cortisol	953.2	1127.3	574.7	152.9	46.7	23.3

Same reference values of Table 1.

**Table 3:** Bilateral inferior petrosal sinus sampling.

Time (minutes)	Inferior petrosal sinus		Peripheral
	Right	Left	Blood
−5 minutes	209.0 ng/L	206.0 ng/L	186.0 ng/L
0 minutes	213.0 ng/L	207.0 ng/L	190.0 ng/L
2-3 minutes	215.0 ng/L	203.0 ng/L	190.0 ng/L
5 minutes	210.0 ng/L	212.0 ng/L	191.0 ng/L
10 minutes	222.0 ng/L	219.0 ng/L	206.0 ng/L
15 minutes	222.0 ng/L	226.0 ng/L	216.0 ng/L

**Table 4:** CRH stimulation test.

Time (minutes)	ACTH (ng/L)	Cortisol (*μ*g/dL)
−15	253.00	34.1
0	240.00	32.4
15	255.00	32.4
30	248.00	33.2
60	239.00	33.4
90	232.00	33.3
120	221.00	33.3

## Abbreviations

ACTH: Adrenocorticotropic hormone
CT: Computed tomography
MRI: Magnetic resonance imaging
^18^-FDG-PET: ^18^-F-Fluorodeoxyglucose positron emission tomography
CRH: Corticotropin-releasing hormone
HPF: High powered fields
SRS: Somatostatin receptor scintigraphy.
